# Differential Telomere Shortening in Blood versus Arteries in an Animal Model of Type 2 Diabetes

**DOI:** 10.1155/2015/153829

**Published:** 2015-08-06

**Authors:** Samira Tajbakhsh, Kamelya Aliakbari, Damian J. Hussey, Karen M. Lower, Anthony J. Donato, Elke M. Sokoya

**Affiliations:** ^1^Discipline of Biotechnology, School of Medicine, Flinders University, Bedford Park, SA 5042, Australia; ^2^Discipline of Surgery, School of Medicine, Flinders University, Bedford Park, SA 5042, Australia; ^3^Discipline of Haematology, School of Medicine, Flinders University, Bedford Park, SA 5042, Australia; ^4^Department of Internal Medicine, University of Utah, Salt Lake City, UT 84132, USA; ^5^Discipline of Human Physiology, School of Medicine, Flinders University, Bedford Park, SA 5042, Australia

## Abstract

Vascular dysfunction is an early feature of diabetic vascular disease, due to increased oxidative stress and reduced nitric oxide (NO) bioavailability. This can lead to endothelial cell senescence and clinical complications such as stroke. Cells can become senescent by shortened telomeres and oxidative stress is known to accelerate telomere attrition. Sirtuin 1 (SIRT1) has been linked to vascular health by upregulating endothelial nitric oxide synthase (eNOS), suppressing oxidative stress, and attenuating telomere shortening. Accelerated leukocyte telomere attrition appears to be a feature of clinical type 2 diabetes (T2D) and therefore the telomere system may be a potential therapeutic target in preventing vascular complications of T2D. However the effect of T2D on vascular telomere length is currently unknown. We hypothesized that T2D gives rise to shortened leukocyte and vascular telomeres alongside reduced vascular SIRT1 expression and increased oxidative stress. Accelerated telomere attrition was observed in circulating leukocytes, but not arteries, in T2D compared to control rats. T2D rats had blunted arterial SIRT1 and eNOS protein expression levels which were associated with reduced antioxidant defense capacity. Our findings suggest that hyperglycemia and a deficit in vascular SIRT1 *per se* are not sufficient to prematurely shorten vascular telomeres.

## 1. Introduction

Type 2 diabetes (T2D) is one of the leading causes of mortality worldwide, largely due to vascular complications including myocardial infarction and stroke [1, 2]. Endothelial function, a key determinant of vascular health, is impaired in the diabetic vasculature primarily due to reduced nitric oxide (NO) bioavailability that is brought about by a combination of uncoupling and inhibition of endothelial nitric oxide synthase (eNOS) and an excessive production of reactive oxygen species (ROS) [3]. Loss of NO bioactivity, leading to endothelial dysfunction, is known to predispose patients to developing secondary complications such as stroke [4]. Therefore understanding the mechanisms associated with vascular dysfunction is critical to the development of therapeutic targets in the hope of preventing T2D-related morbidity.

The NAD^+^-dependent deacetylase, sirtuin 1 (SIRT1), is abundantly expressed in the vasculature [5] and deacetylates a number of substrates that are key modulators of vascular function including eNOS [6] and FOXO1 [7]. SIRT1 has been reported to be downregulated in peripheral blood mononuclear cells of obese humans [8] and in the liver and pancreas of rodents on a high-fat diet [9]. The effect of T2D on microvascular SIRT1 expression is less clear. High-fat feeding resulted in a significant decrease in mouse aorta SIRT1 protein expression [10], while no changes were seen in the cerebral vasculature in obese Zucker rats [11].

SIRT1 interacts with specific double-stranded repeat sequences of DNA at the extremities of chromosomes [5′-(TTAGGG)_n_-3′], known as telomeres [12]. During each round of cell division, telomeres shorten until they reach a critical length [13]. At this point, the cell can no longer replicate and it becomes either senescent or apoptotic, thereby creating an accelerated aging phenotype and increasing risk of secondary disease [14]. Telomere shortening can be prematurely accelerated* in vitro* in the face of oxidative stress [15], a hallmark of T2D [16]. Therefore restoring telomere health may be an important therapeutic target in preventing vascular complications of T2D. Although epidemiological studies have suggested an association between T2D and shortened leukocyte telomeres [17, 18], a direct cause and effect relationship has never been established. Moreover it is not known whether vascular telomeres have accelerated shortening in T2D.

## 2. Materials and Methods

### 2.1. Animal Model of Type 2 Diabetes

All animal procedures were carried out in accordance with the Australian Code for the Responsible Conduct of Research and were approved by the Flinders University Animal Ethics Committee. Male hooded Wistar rats (*n* = 17) were bred and housed under controlled room temperature with a 12 : 12 hour light-dark cycle in the School of Medicine Animal Care Facility at Flinders University.

**Figure 1:**
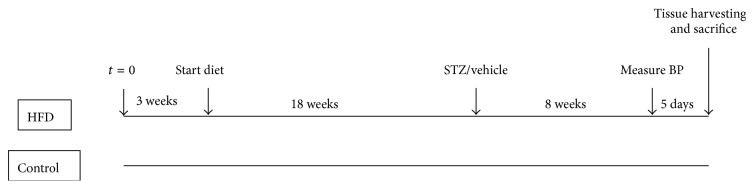
Timeline depicting the initiation of the assigned diet (high-fat or control diet), administration of streptozotocin or vehicle, blood pressure measurement, and tissue harvesting followed by termination.

After weaning at three weeks of age, rats were randomly assigned into two groups: diabetic and control. The diabetic group (*n* = 9; T2D) was placed on a high-fat diet which consisted of 45 kcal% fat, 35 kcal% carbohydrate, and 20 kcal% protein (D12451; Open Source Diet). The chow was custom-made by Gordon's specialty stockfeed (Yanderra, Australia). The remaining group (*n* = 8; control) was maintained on a control diet which contained 10 kcal% fat, 70 kcal% carbohydrate, and 20 kcal% protein (D12450H; Open Source Diet). Animals had free access to water and their assigned diets. After 18 weeks on the diet regimen, the diabetic group was given an intraperitoneal injection of streptozotocin (30 mg/kg body weight) dissolved in saline while the control group was injected with the same volume of vehicle. Both groups were maintained on their respective diets for an additional eight weeks. A timeline is shown in Figure 1. Previous studies have shown that a high-fat feeding followed by a low dose of streptozotocin induces a mild impairment of insulin secretion, similar to that seen in late stage T2D [19].

### 2.2. Measurement of Blood Pressure

Five days prior to sacrifice, rats were anesthetized with 3% isoflurane and their body temperature was maintained on a heating pad at 37°C. Blood pressure was measured using MLT125/R pulse transducer/pressure cuff and IN125 NIBP controller (AD Instruments, Australia) and recorded using PowerLab software (AD Instruments, Australia). Systolic pressure was determined from the average of three consecutive readings obtained from each rat.

### 2.3. Sample Collection

On the day of sacrifice, animals were weighed and anesthetized with isoflurane. Blood glucose was measured from the tail vein using an Accu-Chek Performa glucometer and test strips (Roche Diagnostics, Indianapolis, IN, USA). The animals were then decapitated using a rodent guillotine and trunk blood obtained for measurement of serum insulin and free fatty acids, plasma triglycerides, and C-reactive protein (CRP) and for extraction of DNA using a DNeasy blood and tissue kit (Qiagen) according to the manufacturer's instructions. The DNA concentration of each extraction was measured in triplicate using a NanoDrop 2000 (Thermo Scientific).

The left and right femoral artery were excised from animals, cleaned of connective tissue, and washed to remove blood. The size of each artery was 0.9 cm in length, 2 mm in en face width, and approximately 1 mg in weight. Arteries were immediately snap frozen in liquid nitrogen and stored at −80°C. DNA was extracted using a QIAamp DNA micro kit (Qiagen) according to the manufacturer's instructions and stored at −80°C until analysis.

The brain was removed and the middle cerebral arteries were carefully harvested and pooled into 180 *µ*L modified radioimmunoprecipitation (RIPA) buffer containing 50 mM Tris, pH 7.8 with 150 mM NaCl, 1% Triton X-100, 0.5% Nonidet P-40, 0.25% sodium deoxycholate, and 1 mM PMSF and protease inhibitor cocktail (Roche, Indianapolis, IN, USA). Total protein was extracted as previously described [20] and stored at −80°C.

### 2.4. Immunoblotting

Total protein was measured using EZQ Protein Quantitation (Roche Diagnostics, Indianapolis, IN, USA). Cerebral artery lysate was then combined with Laemmli sample buffer and heated to 95°C for 5 mins. Equal amounts of protein (18 micrograms) were loaded onto each lane of a stain-free gel (Bio-Rad Laboratories, Hercules, CA, USA) alongside Dual Color Precision Plus protein standard (Bio-Rad Laboratories). Total protein loaded on the gel and then transferred to the low fluorescence polyvinylidene difluoride (PVDF) membrane was imaged (ChemiDoc MP imager, Bio-Rad Laboratories) and analysed using Image Lab 4.0.1 software (Bio-Rad Laboratories). Previous studies have shown that this method of protein normalization is superior to that of either total protein stains (e.g., Sypro Ruby, Amido Black, and Coomassie Blue) or antibody loading controls [21].

Membranes were probed with an antibody directed against either SIRT1 (Abcam), eNOS (BD Transduction Labs), Nox2 (BD Transduction Laboratories), manganese superoxide dismutase (MnSOD; Millipore), p66^Shc^ (Millipore), or 3-nitrotyrosine (Abcam) overnight at 4°C. The next day, membranes were washed and incubated with the appropriate secondary antibody (Jackson ImmunoResearch). Bands were visualized using enhanced chemiluminescence, captured with a digital acquisition system (Fujifilm, Japan) and quantified (Carestream Molecular Imaging software, Rochester, NT, USA). Densitometry results are expressed relative to total protein and normalized to the control group.

### 2.5. Singleplex Telomere qPCR Assay

Mean telomere length was measured using a singleplex assay similar to that described by Cawthon [22]. The telomere and single-copy gene (*β*-actin) were amplified in separate singleplex reactions because the efficiency of each amplicon was optimal in different mastermixes (assessed in pilot studies). The telomere primers were used as previously described by Cawthon [23] and used at a concentration of 900 nM. The single-copy gene (*β*-actin) primer sequences (written 5′ → 3′) were *β*-actin-F, AGG TCA TCA CTA TCG GCA ATG A, and *β*-actin-R, GAG ACT ACA ACT TAC CCA GGA AGG AA, and used at a final concentration of 2 *µ*M.

Each qPCR reaction was conducted in a total volume of 20 *µ*L using 384-well plates and performed on a ViiA 7 real-time PCR instrument (Life Technologies, Foster City, CA). The telomere assay was amplified in a custom-made mastermix as described by Cawthon [23] and the *β*-actin assay was amplified in Power SYBR green PCR mastermix (Life Technologies).

For the telomere assay, a standard curve was run alongside the experimental samples consisting of a calibrator DNA sample which was diluted serially by 5-fold to produce five concentrations of DNA ranging from 515 to 0.824 ng/mL. For the *β*-actin assay, a standard curve was run alongside the experimental samples consisting of the same calibrator DNA sample which was diluted serially by 5-fold to produce five concentrations of DNA ranging from 5150 to 8.24 ng/mL. The calibrator was rat DNA extracted from either whole blood or femoral artery.

### 2.6. Telomere Data Analysis

The amount of telomere was calculated as *T* = *E* ^(Cq_calibrator_ − Cq_sample_)^, where *E* was the efficiency of the telomere primer calculated from the standard curve, Cq_calibrator_ was the Cq of the 1 : 5 dilution on the standard curve, and Cq_sample_ was the Cq of the experimental sample. The amount of *β*-actin (reference gene) was calculated as *S* = *E* ^(Cq_calibrator_ − Cq_sample_)^, where *E* was the efficiency of the *β*-actin primer calculated from the standard curve, Cq_calibrator_ was the Cq of the 1 : 5 dilution on the standard curve, and Cq_sample_ was the Cq of the experimental sample. Relative telomere length (*T*/*S*) was estimated as the ratio between the amount of telomere (*T*) and reference gene (*S*).

### 2.7. Drugs and Reagents

Streptozotocin was purchased from Sigma. Fresh streptozotocin stock solutions of 50 mg/mL were made in saline. The *β*-actin and telomere primers were synthesized by GeneWorks (Thebarton, SA, Australia) and dissolved in water for injection (GlaxoSmithKline).

### 2.8. Statistical Analysis and Power

All data are expressed as mean ± standard error of the mean. Statistical comparisons were performed using GraphPad InStat version 3 software (GraphPad software, La Jolla, CA, USA) using an unpaired *t*-test. Statistical significance was accepted at *P* < 0.05. The initial power analysis was performed based on previous telomere length studies in rodents [24, 25]. The experiment was powered at 80% to detect a 20% change in telomere length, assuming a two-tailed alpha level of 5%.

## 3. Results

### 3.1. Phenotypic Assessment of Animals Fed a High-Fat Diet followed by a Low Dose of Streptozotocin

Rats fed a high-fat diet for 18 weeks weighed significantly more than age-matched control rats fed a control diet (*P* = 0.029; see Table 1). Animals then received either a low dose of streptozotocin or vehicle and the diet regimen was continued for another eight weeks. One rat died just prior to streptozotocin injection and two rats died the following week after streptozotocin injection. Systolic blood pressure was similar in both T2D rats and control rats (*P* = 0.16; 113 ± 8 mmHg versus 105 ± 5 mmHg, resp.). On the day of sacrifice, T2D rats weighed significantly less than the control group (*P* = 0.0001; 358 ± 12 g versus 432 ± 7 g, resp.).

Fasting plasma glucose levels were significantly higher in T2D rats compared to control rats (*P* < 0.0001; 24.2 ± 1.3 mM versus 8.6 ± 0.3 mM, resp.). Circulating free fatty acid concentration was significantly elevated in T2D rats compared to control rats (*P* = 0.012; 0.51 ± 0.06 mM versus 0.34 ± 0.02 mM, resp.). Plasma insulin concentration was significantly lower in T2D rats compared to the control rats (*P* = 0.0028; 0.5 ± 0.1 ng/mL versus 2.8 ± 0.5 ng/mL, resp.), a reflection of streptozotocin-induced pancreatic *β*-cell dysfunction. As a marker of systemic inflammation, plasma CRP was significantly elevated in T2D compared to control rats (*P* < 0.0001; see Table 1).

### 3.2. Mean Telomere Length in Femoral Artery and Whole Blood

**Figure 2:**
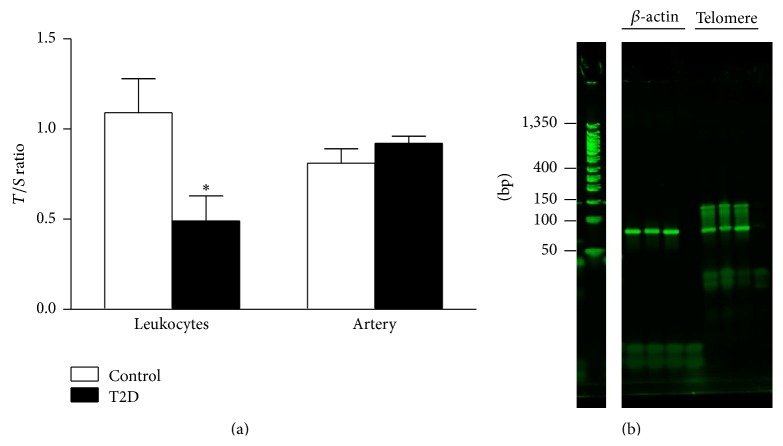
(a) Leukocyte *T*/*S* ratio was significantly lower in T2D rats compared to control rats (*P* = 0.034; *t*-test). Femoral artery *T*/*S* ratio was comparable between T2D and control rats (*P* = 0.28). (b) qPCR products for rat *β*-actin and telomere primers. Lane 1: DNA ladder; lanes 2 and 6: rat leukocyte calibrator DNA; lanes 3 and 7: control rat leukocyte DNA; lanes 4 and 8: T2D rat leukocyte DNA; lanes 5 and 9: no template control (H_2_O).

As shown in Figure 2(a), arterial telomere length was comparable between T2D rats and control rats (*P* = 0.28; 0.92 ± 0.04 versus 0.81 ± 0.08, resp.). However leukocyte telomere length was significantly shorter in T2D compared to the control group (*P* = 0.034; 0.49 ± 0.14 versus 1.09 ± 0.19, resp.). The percent efficiency of *β*-actin and telomere primers ranged between 92 and 102%. The PCR amplification products were visualized by agarose gel electrophoresis, ethidium bromide staining, and UV transillumination (see Figure 2(b)). The *β*-actin PCR product was observed at the expected size of 81 base pairs while the telomere PCR product showed a main product at 82 base pairs (the sum of the length of the two telomere primers) and a smear to 150 base pairs, reflecting longer PCR products due to staggered annealing of the primers. This is consistent with other reports in the literature [22, 26].

### 3.3. Vascular Protein Expression

**Figure 3:**
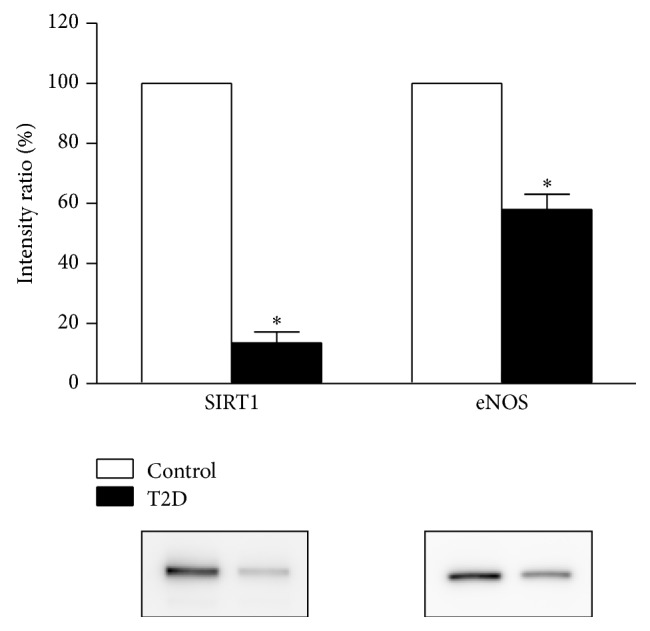
Arterial protein expression levels of SIRT1 and eNOS were decreased in T2D rats compared to control rats (*n* = 3 each; *P* = 0.0082 and *P* = 0.011, resp.; *t*-test). Representative immunoblots are shown below the summary data. Relative protein levels were calculated as the ratio of the intensity of the chemiluminescent bands to the intensity of the total protein loaded on the gel and are expressed as a percentage of the values measured in control rats.

**Figure 4:**
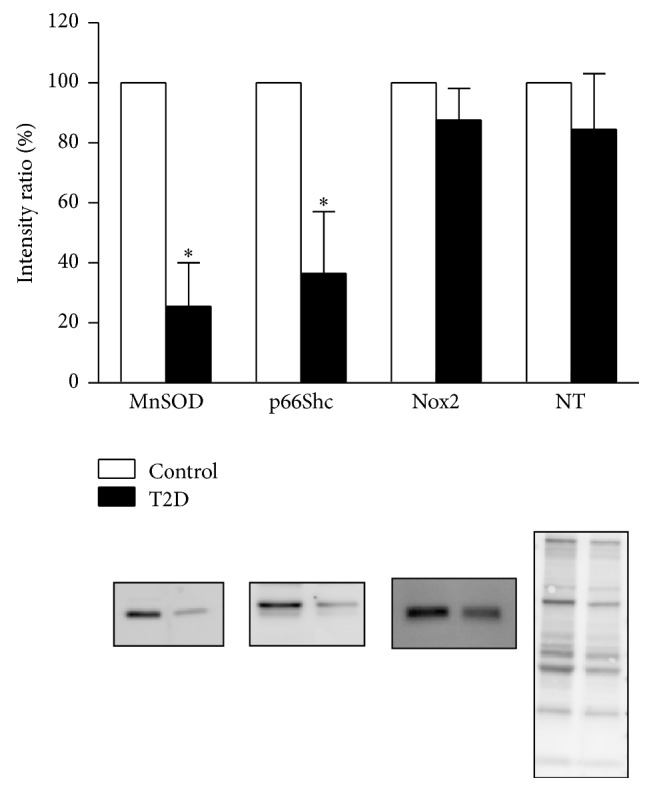
Arterial protein expression levels of MnSOD and p66Shc, but not Nox2 or 3-NT, were significantly decreased in T2D rats compared to control rats (*n* = 3 each; *P* = 0.039 and *P* = 0.028, resp.;* t*-test). Representative immunoblots are shown below the summary data. Relative protein levels were calculated as the ratio of the intensity of the chemiluminescent bands to the intensity of the total protein loaded on the gel and are expressed as a percentage of the values measured in control rats.

Vascular SIRT1 and eNOS protein expression were significantly lower in T2D rats compared to their control counterparts (*P* = 0.0082 and *P* = 0.011, resp.; see Figure 3). MnSOD and p66Shc protein levels were significantly lower in T2D rats compared to control rats (*P* = 0.039 and *P* = 0.028, resp.); however total 3-nitrotyrosine-containing proteins and Nox2 were comparable between groups (Figure 4).

## 4. Discussion

There are two major findings of the present study. Firstly, we have demonstrated that T2D causes selective accelerated telomere shortening in circulating leukocytes but preserved telomere length in arteries. To our knowledge, this is the first direct evidence of leukocyte telomere shortening in T2D. Secondly, although hyperglycemia leads to blunted SIRT1 [27] and telomere shortening [28], we have shown* in vivo* that hyperglycemia and a deficit in vascular SIRT1* per se* are not sufficient to prematurely shorten vascular telomeres.

### 4.1. Phenotype of T2D Rats

Here we have shown that rats gained significantly more weight after an 18-week high-fat diet regimen (see Table 1). Injection of a low dose of streptozotocin significantly increased blood glucose levels in T2D rats, which is consistent with other reports [29]. Circulating insulin levels were significantly decreased, a feature of clinical late stage T2D when the pancreatic beta cells become impaired and insulin secretion is reduced [30]. The animals also showed evidence of dyslipidemia that is in line with elevated plasma free fatty acids observed in human T2D [31] that has been linked to the onset of insulin resistance [32]. The T2D rats in our study had significantly elevated levels of CRP, confirming that this animal model is a good representation of T2D in humans which is associated with prolonged low-grade inflammation [33].

### 4.2. Leukocyte Telomere Length Is Shorter in T2D

We found that leukocyte telomere length is shorter in T2D rats compared to control rats. Our findings are in line with accumulating clinical studies showing a correlation between T2D and shorter leukocyte telomeres [17, 18, 34, 35]. One possible explanation for the shorter leukocyte telomeres in T2D is that it reflects increased leukocyte turnover due to the systemic low-grade inflammation. However clinical studies have failed to show a significant inverse correlation between leukocyte telomere length and markers of inflammation [35–37]. Tentolouris and colleagues [38] have previously reported a correlation between telomere length and nitrosative stress. Taken together, we speculate that the observed leukocyte telomere attrition may be due, at least in part, to nitrosative stress which is absent within the vasculature in this animal model of T2D (see below).

### 4.3. Vascular Telomere Length Is Comparable in T2D and Controls

Shortened vascular telomere length has been previously documented in human coronary heart disease [39], atherosclerosis [40], and abdominal aortic aneurysms [41]. Because vascular biopsies cannot be taken from obese or T2D patients without overt vascular pathology, leukocyte telomere measurements have been used as a surrogate for predicting vascular disease risk [42]. We have shown for the first time that arterial telomere length is preserved in T2D rats compared to control rats. Although SIRT1 depletion [43] and hyperglycemia [28] have previously been shown to independently accelerate telomere shortening, our results have shown that hyperglycemia and blunted vascular SIRT1* in vivo* were not sufficient to prematurely shorten arterial telomeres. Our experiments were sufficiently powered at 80% to detect a 20% change in telomere length. Therefore a smaller change in telomere length would not have been detected and as a result we cannot rule out the possibility that there are more subtle changes in telomere length within the T2D vasculature. Nevertheless our data suggests that leukocyte telomere length may not be an optimal surrogate for vascular telomere length in T2D.

### 4.4. Vascular SIRT1 and eNOS Protein Expression Are Reduced in T2D

SIRT1 protein expression was significantly decreased in resistance-sized vessels harvested from T2D rats compared to control rats on a normal diet (standard chow). Our findings are in line with studies showing a decline in SIRT1 levels at the level of the macrovasculature (aorta) after six months of high-fat feeding [10]. However it is important to assess protein expression within the resistance-sized vessels, since these vessels are fundamental to the control of blood flow [44]. We have now confirmed for the first time that SIRT1 protein expression is blunted within the microvasculature in T2D.

The mechanisms leading to a decline in SIRT1 expression in T2D are incompletely understood. SIRT1 activity has been shown to be controlled by both nutritional status and the cellular redox status (NAD^+^/NADH ratio). In a nutrient rich environment, NAD^+^/NADH ratio is blunted due to an increased rate of reduction of NAD^+^ to NADH, leading to a decrease in SIRT1 activity [10]. Endothelial progenitor cells [45], human umbilical vein endothelial cells [27], and mouse brain endothelial cells (Sokoya et al., unpublished findings) cultured in high glucose have lower levels of SIRT1 protein expression. Recent studies have also shown that oxidative stress directly inhibits SIRT1 activity via oxidative posttranslational modifications [46]. Therefore, both a reduction in cellular redox status and an increase in oxidant stress may be working together to reduce vascular SIRT1 expression.

NO is an important signalling molecule in the vascular system. The production of endothelial-derived NO is key in mediating relaxation of the cerebral vasculature [47]. Our study has found that T2D leads to significantly lower eNOS protein levels within cerebral arteries. This may be mediated in part by CRP, which has been shown to directly decrease eNOS protein expression and activity [48] and/or hyperglycemia that promotes eNOS glycosylation thereby reducing the active form of NO [49]. A reduction in total eNOS production suggests that NO bioavailability may be reduced, potentially leading to blunted endothelial-mediated relaxations, which have previously been shown in this model [50].

### 4.5. Vascular Antioxidant Protein Expression Is Reduced in T2D

Our observation that vascular MnSOD protein expression is blunted in T2D suggests that the antioxidant pathway is being overwhelmed, presumably due to an increase in superoxide production within the vasculature. This is consistent with other data showing downregulation of MnSOD in the vasculature after high-fat feeding [51].

Vascular p66Shc protein expression was also significantly lower in T2D rats compared to controls. As a redox mitochondrial enzyme, p66Shc has been shown to generate mitochondrial ROS (hydrogen peroxide) [52]. However more recent studies have localized p66Shc to the cytoplasm where it is regulated by the redox sensitive transcription factor, NFE2-related factor 2 (Nrf2) [53]. Nrf2 regulates the antioxidant response element (ARE/EpRE) mediated expression of antioxidant enzymes. In this way, cytosolic p66Shc serves as an antioxidant protein. Our finding of blunted vascular p66Shc protein levels in T2D is in accordance with a recent report of reduced Nrf2 signaling in mouse brain after high-fat feeding [54].

Interestingly, SIRT1 itself has been shown to play an important role in regulating antioxidant gene expression in endothelial cells by enhancing the stability of the FoxO3a/PGC-1*α* complex, which promotes antioxidant gene expression [7]. In this way, blunted vascular SIRT1 in T2D could mediate, at least in part, the observed decrease in antioxidant protein expression.

### 4.6. Source of Vascular Superoxide

Vascular NADPH oxidases are one of the major sources of ROS in the cardiovascular system [55]. In our study, we examined a subunit of NADPH oxidase, Nox2, which has previously shown to be significantly increased in mouse aorta after high-fat feeding [56]. However, in our animal model of T2D, Nox2 levels were uncompromised in cerebral artery homogenates. Nevertheless there are additional sources of superoxide including cyclooxygenase [57], xanthine oxidase [58], and other NADPH oxidases such as Nox1 and Nox5 [55].

### 4.7. Nitrosative Stress

Superoxide can react with NO to form peroxynitrite which in turn promotes nitration of tyrosine residues in proteins leading to nitrotyrosine-containing proteins. In the present study, we found that the abundance of nitrotyrosine-modified proteins was comparable in cerebral artery lysate from T2D rats compared to control rats. This is in contrast to studies in mouse aorta [59], rat aorta [60], pig coronary artery [61], and rat epineurial arterioles [62] that have shown increased nitrotyrosine expression following high-fat feeding. Taken together, our data suggest that, although antioxidant defence capacity is reduced, there appears to be no vascular nitrosative stress in our experimental model of T2D.

### 4.8. Concluding Remarks

A number of caveats need to be recognised here in terms of interpretation of our data. Firstly, markers of oxidative and nitrosative stress were measured within the cerebral vasculature while telomere length was measured in DNA extracted from the peripheral vasculature. Ideally, telomere length would have been measured within cerebral arteries; however this was impossible given the small size of rodent cerebral arteries. Secondly, the femoral artery comprises a single layer of endothelial cells and a few layers of smooth muscle cells. Therefore the smooth muscle component largely outweighs the endothelial component. If there are changes in endothelial cell telomere length in the absence of smooth muscle cell changes, these would not be detected. Thirdly, the qPCR assay as used in the present study measures mean telomere length. There is evidence that a cell may be triggered to enter senescence by a single critically short telomere [63]. Therefore it remains a possibility that there is telomere dysfunction within the T2D vasculature, driven by a small number of critically short telomeres. Future studies could address this possibility using high-throughput quantitative fluorescence* in situ* hybridization [64].

In summary, we have shown for the first time that leukocyte telomere length does not predict arterial telomere length in T2D rats. Our findings highlight the importance of measuring telomere dynamics in tissues other than circulating immune cells.

## Figures and Tables

**Table 1:** Phenotype of control (*n* = 8) and T2D (*n* = 6) rats. Values are presented as mean ± standard error of the mean. *∗* indicates *P* < 0.05 between T2D and control.

	Control	T2D	*P* value
Weight at injection (g)	387 ± 5	413 ± 9	0.029^*∗*^
Final weight (g)	432 ± 7	358 ± 12	0.0001^*∗*^
Systolic pressure (mmHg)	105 ± 5	113 ± 5	0.15
Glucose (mM)	8.6 ± 0.3	24.2 ± 1.3	<0.0001^*∗*^
Insulin (ng/mL)	2.8 ± 0.5	0.5 ± 0.1	0.0028^*∗*^
Triglycerides (mM)	1.4 ± 0.1	2.0 ± 0.4	0.14
Free fatty acids (mM)	0.34 ± 0.02	0.51 ± 0.06	0.012^*∗*^
CRP (ng/mL)	16 ± 0.7	263 ± 23	<0.0001^*∗*^

## References

[1] Winer N., Sowers J. R. (2004). Epidemiology of diabetes. *Journal of Clinical Pharmacology*.

[2] Preis S. R., Pencina M. J., Hwang S.-J. (2009). Trends in cardiovascular disease risk factors in individuals with and without diabetes mellitus in the Framingham Heart Study. *Circulation*.

[3] Ding H., Triggle C. R. (2010). Endothelial dysfunction in diabetes: multiple targets for treatment. *Pflugers Archiv European Journal of Physiology*.

[4] Miller A. A., Budzyn K., Sobey C. G. (2010). Vascular dysfunction in cerebrovascular disease: mechanisms and therapeutic intervention. *Clinical Science*.

[5] Tajbakhsh N., Sokoya E. M. (2012). Regulation of cerebral vascular function by sirtuin 1. *Microcirculation*.

[6] Mattagajasingh I., Kim C.-S., Naqvi A. (2007). SIRT1 promotes endothelium-dependent vascular relaxation by activating endothelial nitric oxide synthase. *Proceedings of the National Academy of Sciences of the United States of America*.

[7] Olmos Y., Valle I., Borniquel S. (2009). Mutual dependence of Foxo3a and PGC-1*α* in the induction of oxidative stress genes. *Journal of Biological Chemistry*.

[8] De Kreutzenberg S. V., Ceolotto G., Papparella I. (2010). Downregulation of the longevity-associated protein sirtuin 1 in insulin resistance and metabolic syndrome: potential biochemical mechanisms. *Diabetes*.

[9] Chen Y.-R., Fang S.-R., Fu Y.-C., Zhou X.-H., Xu M.-Y., Xu W.-C. (2010). Calorie restriction on insulin resistance and expression of SIRT1 and SIRT4 in rats.. *Biochemistry and Cell Biology*.

[10] Zhang Q.-J., Wang Z., Chen H.-Z. (2008). Endothelium-specific overexpression of class III deacetylase SIRT1 decreases atherosclerosis in apolipoprotein E-deficient mice. *Cardiovascular Research*.

[11] Tajbakhsh N., Sokoya E. M. (2013). Sirtuin 1 is upregulated in young obese Zucker rat cerebral arteries. *European Journal of Pharmacology*.

[12] Palacios J. A., Herranz D., De Bonis M. L., Velasco S., Serrano M., Blasco M. A. (2010). SIRT1 contributes to telomere maintenance and augments global homologous recombination. *The Journal of Cell Biology*.

[13] Blasco M. A. (2007). The epigenetic regulation of mammalian telomeres. *Nature Reviews Genetics*.

[14] Wang C.-Y., Kim H.-H., Hiroi Y. (2009). Obesity increases vascular senescence and susceptibility to ischemic injury through chronic activation of Akt and mTOR. *Science Signaling*.

[15] von Zglinicki T. (2002). Oxidative stress shortens telomeres. *Trends in Biochemical Sciences*.

[16] Giacco F., Brownlee M. (2010). Oxidative stress and diabetic complications. *Circulation Research*.

[17] Zee R. Y. L., Castonguay A. J., Barton N. S., Germer S., Martin M. (2010). Mean leukocyte telomere length shortening and type 2 diabetes mellitus: a case-control study. *Translational Research*.

[18] Murillo-Ortiz B., Albarrán-Tamayo F., Arenas-Aranda D. (2012). Telomere length and type 2 diabetes in males, a premature aging syndrome. *Aging Male*.

[19] Srinivasan K., Viswanad B., Asrat L., Kaul C. L., Ramarao P. (2005). Combination of high-fat diet-fed and low-dose streptozotocin-treated rat: a model for type 2 diabetes and pharmacological screening. *Pharmacological Research*.

[20] Tajbakhsh N., Sokoya E. M. (2014). Compromised endothelium-dependent hyperpolarization-mediated dilations can be rescued by NS309 in obese Zucker rats. *Microcirculation*.

[21] Colella A. D., Chegenii N., Tea M. N., Gibbins I. L., Williams K. A., Chataway T. K. (2012). Comparison of Stain-Free gels with traditional immunoblot loading control methodology. *Analytical Biochemistry*.

[22] Cawthon R. M. (2002). Telomere measurement by quantitative PCR. *Nucleic Acids Research*.

[23] Cawthon R. M. (2009). Telomere length measurement by a novel monochrome multiplex quantitative PCR method. *Nucleic Acids Research*.

[24] da Luz P. L., Tanaka L., Brum P. C. (2012). Red wine and equivalent oral pharmacological doses of resveratrol delay vascular aging but do not extend life span in rats. *Atherosclerosis*.

[25] Raymond A. R., Hodson B., Woodiwiss A. J., Norton G. R., Brooksbank R. L. (2013). Telomere length and adrenergic-induced left ventricular dilatation and systolic chamber dysfunction in rats. *European Journal of Applied Physiology*.

[26] Lee D.-C., Im J.-A., Kim J.-H., Lee H.-R., Shim J.-Y. (2005). Effect of long-term hormone therapy on telomere length in postmenopausal women. *Yonsei Medical Journal*.

[27] Orimo M., Minamino T., Miyauchi H., Tateno K., Okada S., Komuro I. (2009). Protective role of SIRT1 in diabetic vascular dysfunction. *Arteriosclerosis, Thrombosis, and Vascular Biology*.

[28] Matsui-Hirai H., Hayashi T., Yamamoto S. (2011). Dose-dependent modulatory effects of insulin on glucose-induced endothelial senescence in vitro and in vivo: a relationship between telomeres and nitric oxide. *Journal of Pharmacology and Experimental Therapeutics*.

[29] Davidson E. P., Coppey L. J., Calcutt N. A., Oltman C. L., Yorek M. A. (2010). Diet-induced obesity in Sprague-Dawley rats causes microvascular and neural dysfunction. *Diabetes/Metabolism Research and Reviews*.

[30] Lebovitz H. E., Banerji M. A. (2004). Treatment of insulin resistance in diabetes mellitus. *European Journal of Pharmacology*.

[31] Reaven G. M., Hollenbeck C., Jeng C.-Y., Wu M. S., Chen Y.-D. I. (1988). Measurement of plasma glucose, free fatty acid, lactate, and insulin for 24 h in patients with NIDDM. *Diabetes*.

[32] Boden G. (1997). Role of fatty acids in the pathogenesis of insulin resistance and NIDDM. *Diabetes*.

[33] Donath M. Y., Shoelson S. E. (2011). Type 2 diabetes as an inflammatory disease. *Nature Reviews Immunology*.

[34] Adaikalakoteswari A., Balasubramanyam M., Mohan V. (2005). Telomere shortening occurs in Asian Indian Type 2 diabetic patients. *Diabetic Medicine*.

[35] Salpea K. D., Talmud P. J., Cooper J. A. (2010). Association of telomere length with type 2 diabetes, oxidative stress and UCP2 gene variation. *Atherosclerosis*.

[36] Olivieri F., Lorenzi M., Antonicelli R. (2009). Leukocyte telomere shortening in elderly Type2DM patients with previous myocardial infarction. *Atherosclerosis*.

[37] Testa R., Olivieri F., Sirolla C. (2011). Leukocyte telomere length is associated with complications of type 2 diabetes mellitus. *Diabetic Medicine*.

[38] Tentolouris N., Nzietchueng R., Cattan V. (2007). White blood cells telomere length is shorter in males with type 2 diabetes and microalbuminuria. *Diabetes Care*.

[39] Ogami M., Ikura Y., Ohsawa M. (2004). Telomere shortening in human coronary artery diseases. *Arteriosclerosis, Thrombosis, and Vascular Biology*.

[40] Matthews C., Gorenne I., Scott S. (2006). Vascular smooth muscle cells undergo telomere-based senescence in human atherosclerosis: effects of telomerase and oxidative stress. *Circulation Research*.

[41] Wilson W. R. W., Herbert K. E., Mistry Y. (2008). Blood leucocyte telomere DNA content predicts vascular telomere DNA content in humans with and without vascular disease. *European Heart Journal*.

[42] Fyhrquist F., Saijonmaa O. (2012). Telomere length and cardiovascular aging. *Annals of Medicine*.

[43] De Bonis M. L., Ortega S., Blasco M. A. (2014). SIRT1 is necessary for proficient telomere elongation and genomic stability of induced pluripotent stem cells. *Stem Cell Reports*.

[44] Cipolla M. J. (2009). *Integrated Systems Physiology: From Molecule to Function*.

[45] Balestrieri M. L., Rienzo M., Felice F. (2008). High glucose downregulates endothelial progenitor cell number via SIRT1. *Biochimica et Biophysica Acta*.

[46] Shao D., Fry J. L., Han J. (2014). A redox-resistant sirtuin-1 mutant protects against hepatic metabolic and oxidant stress. *The Journal of Biological Chemistry*.

[47] You J., Johnson T. D., Childres W. F., Bryan R. M. (1997). Endothelial-mediated dilations of rat middle cerebral arteries by ATP and ADP. *The American Journal of Physiology—Heart and Circulatory Physiology*.

[48] Venugopal S. K., Devaraj S., Yuhanna I., Shaul P., Jialal I. (2002). Demonstration that C-reactive protein decreases eNOS expression and bioactivity in human aortic endothelial cells. *Circulation*.

[49] Federici M., Menghini R., Mauriello A. (2002). Insulin-dependent activation of endothelial nitric oxide synthase is impaired by O-linked glycosylation modification of signaling proteins in human coronary endothelial cells. *Circulation*.

[50] Davidson E. P., Coppey L. J., Dake B., Yorek M. A. (2011). Treatment of streptozotocin-induced diabetic rats with alogliptin: effect on vascular and neural complications. *Experimental Diabetes Research*.

[51] Roberts C. K., Barnard R. J., Sindhu R. K., Jurczak M., Ehdaie A., Vaziri N. D. (2006). Oxidative stress and dysregulation of NAD(P)H oxidase and antioxidant enzymes in diet-induced metabolic syndrome. *Metabolism: Clinical and Experimental*.

[52] Giorgio M., Migliaccio E., Orsini F. (2005). Electron transfer between cytochrome c and p66Shc generates reactive oxygen species that trigger mitochondrial apoptosis. *Cell*.

[53] Miyazawa M., Tsuji Y. (2014). Evidence for a novel antioxidant function and isoform-specific regulation of the human p66Shc gene. *Molecular Biology of the Cell*.

[54] Morrison C. D., Pistell P. J., Ingram D. K. (2010). High fat diet increases hippocampal oxidative stress and cognitive impairment in aged mice: Implications for decreased Nrf2 signaling. *Journal of Neurochemistry*.

[55] Lassègue B., Griendling K. K. (2010). NADPH oxidases: functions and pathologies in the vasculature. *Arteriosclerosis, Thrombosis, and Vascular Biology*.

[56] Lynch C. M., Kinzenbaw D. A., Chen X. (2013). Nox2-derived superoxide contributes to cerebral vascular dysfunction in diet-induced Obesity. *Stroke*.

[57] Zhu N., Liu B., Luo W. (2014). Vasoconstrictor role of cyclooxygenase-1-mediated prostacyclin synthesis in non-insulin-dependent diabetic mice induced by high-fat diet and streptozotocin. *American Journal of Physiology—Heart and Circulatory Physiology*.

[58] Inkster M. E., Cotter M. A., Cameron N. E. (2007). Treatment with the xanthine oxidase inhibitor, allopurinol, improves nerve and vascular function in diabetic rats. *European Journal of Pharmacology*.

[59] Molnar J., Yu S., Mzhavia N., Pau C., Chereshnev I., Dansky H. M. (2005). Diabetes induces endothelial dysfunction but does not increase neointimal formation in high-fat diet fed C57BL/6J mice. *Circulation Research*.

[60] Bourgoin F., Bachelard H., Badeau M. (2008). Endothelial and vascular dysfunctions and insulin resistance in rats fed a high-fat, high-sucrose diet. *American Journal of Physiology—Heart and Circulatory Physiology*.

[61] Galili O., Versari D., Sattler K. J. (2007). Early experimental obesity is associated with coronary endothelial dysfunction and oxidative stress. *The American Journal of Physiology—Heart and Circulatory Physiology*.

[62] Davidson E. P., Kleinschmidt T. L., Oltman C. L., Lund D. D., Yorek M. A. (2007). Treatment of streptozotocin-induced diabetic rats with AVE7688, a vasopeptidase inhibitor: effect on vascular and neural disease. *Diabetes*.

[63] Hemann M. T., Strong M. A., Hao L.-Y., Greider C. W. (2001). The shortest telomere, not average telomere length, is critical for cell viability and chromosome stability. *Cell*.

[64] Canela A., Vera E., Klatt P., Blasco M. A. (2007). High-throughput telomere length quantification by FISH and its application to human population studies. *Proceedings of the National Academy of Sciences of the United States of America*.

